# A Review on the Potential Bioactive Components in Fruits and Vegetable Wastes as Value-Added Products in the Food Industry

**DOI:** 10.3390/molecules28062631

**Published:** 2023-03-14

**Authors:** Nasir Md Nur ‘Aqilah, Kobun Rovina, Wen Xia Ling Felicia, Joseph Merillyn Vonnie

**Affiliations:** Faculty of Food Science and Nutrition, Universiti Malaysia Sabah, Kota Kinabalu 88400, Sabah, Malaysia

**Keywords:** added value, fruit and vegetable by-products, phytochemical compounds, extraction methods, applications of bioactive compounds

## Abstract

The food production industry is a significant contributor to the generation of millions of tonnes of waste every day. With the increasing public concern about waste production, utilizing the waste generated from popular fruits and vegetables, which are rich in high-added-value compounds, has become a focal point. By efficiently utilizing food waste, such as waste from the fruit and vegetable industries, we can adopt a sustainable consumption and production pattern that aligns with the Sustainable Development Goals (SDGs). This paper provides an overview of the high-added-value compounds derived from fruit and vegetable waste and their sources. The inclusion of bioactive compounds with antioxidant, antimicrobial, and antibrowning properties can enhance the quality of materials due to the high phenolic content present in them. Waste materials such as peels, seeds, kernels, and pomace are also actively employed as adsorbents, natural colorants, indicators, and enzymes in the food industry. Therefore, this article compiles all consumer-applicable uses of fruit and vegetable waste into a single document.

## 1. Introduction

The generation of food waste and by-products is viewed as a significant issue with adverse environmental, economic, and social effects. Food waste minimization is a widespread goal across the country. Despite global efforts, food insecurity remains prevalent. In 2020, the FAO estimated that approximately 721 to 811 million people would experience hunger, with 98% of them residing in developing countries [1]. Approximately 14% of the world’s food supply, valued at around $400 billion, is lost annually between harvest and commercialization [2]. Contrarily, it is estimated that 17% of food is lost or wasted during the retail and consumer stages [3]. Reducing food loss and waste is a crucial strategy for enhancing the efficiency, safety, quality, and sustainability of our food systems. The Food and Agriculture Organization (FAO) is committed to minimizing food waste throughout the production chain from harvest to sale [4]. Additionally, researchers must actively engage in recovering and revaluing crop waste by-products to develop innovations for use in the food industry. This approach can reduce food waste and create more resilient food systems.

During the processing of fruits and vegetables, a wide range of by-products are generated, including a significant quantity of waste in the juice industry. This waste includes leaves, peels, unwanted pulp, seeds, cull fruits, and stones [5,6], which contain high levels of bioactive substances such as polysaccharides (starch, cellulose, and pectin) and proteins [7,8]. Numerous studies have identified a variety of bioactive substances in fruit and vegetable by-products (FVBP)—including vitamin C and phytochemical compounds, such as flavonoids, anthocyanins, carotenoids, and phenolic acids [9,10,11]. Food waste has significant potential for producing edible films and coatings. Food-related biowaste can come from family cafeterias, industrial biowaste, decomposable plants, and botanical garden waste [12]. Packaging made from fruit and vegetable waste is a practical solution for reducing the manufacturing costs of edible films and coatings while adding value to food by-products. Additionally, the performance of the food packaging system can be improved through the different biological qualities of FVBP, such as dietary fibers, antioxidants, and antimicrobials [13,14,15,16].

Extracts of FVBP that contain antioxidant properties have been utilized to create innovative functional food products that are highly sought-after and well-received by consumers [17,18,19]. Furthermore, due to their bioactive properties that offer health benefits to humans, these natural additives can also be utilized in the food, biotechnology, and pharmaceutical industries [20]. However, their practical application is limited by their instability when exposed to regular food processing and storage conditions, such as changes in pH, temperature, light, oxygen, and ions. To address this challenge, an encapsulation method has recently been developed to enhance the stability and water solubility of these bioactive components while protecting them from external factors [21,22]. This review aims to highlight the value-added products that can be derived from fruits and vegetable waste, such as packaging, edible film, natural pigments, encapsulation, and the removal of toxic substances. Additionally, this review provides a comprehensive overview of the various extraction methods used to extract bioactive components from FVBP, including maceration, distillation, microwave-assisted extraction, supercritical fluid extraction, pressurized liquid extraction, pulsed electric field, and ultrasound-assisted extraction.

## 2. Bioactive Components in Fruits and Vegetables Waste

The increasing amount of agroindustrial waste has sparked numerous studies aimed at discovering bioactive compounds, especially in FVBP. These organic waste products contain affordable bioactive compounds (as listed in Table 1) that can be used to create innovative functional food ingredients and additives.

### 2.1. Fruit 

The type of waste produced is generally determined by the fruit used. Fruit peels and seed characteristics are used to categorize them, which helps to determine the processing approach and necessary steps. Berry waste contains anthocyanins, which are responsible for their high total phenolic content and antioxidant activity, which can be affected by the solvent and extraction conditions. For example, blackberry pomace [23], blueberry juice [24]; blackberry, blueberry, and jaboticaba skin [25]; apple peels [26]; strawberry fruit peels [27]; raspberry pomace [28]; and red dragon fruit peels [29] can all be used to extract phenolic acid and anthocyanins compounds. Bioactive components like carotenoids, phenolic acids, and flavonoids can commonly be found in tomato skin [30], pumpkin seeds and peel [31], papaya peel [32], and melon peel [33], and their presence in fruit waste provides antioxidant properties. They can serve as natural preservatives and colorants/indicators in food applications.

El-Beltagi et al. [34] employed agroindustrial waste of dried red beetroot peel extract (DRBP) to evaluate its total phenolic compound, flavonoids, and betalains contents as well as its antioxidant capacity. Additionally, the extract was used as a natural preservative in preserving the quality of Nile tilapia fish filet stored at 5 °C for ten days. Researchers discovered that the DRBP aqueous extract contained compounds with potential antioxidant activity, including a high concentration of TPC (832 mg/100 g), flavonoids (234 mg/100 g), and betalains (535 mg/100 g), making it a suitable natural preservative agent. Fernando et al. [35] recovered the waste materials of red beetroot juice by converting it into high-value chemicals containing betalain, phenolic acid, and flavonoid compounds. The data show that the dried pulp of red beetroot produced a high bioactive yield via ultrasound-assisted extraction. The findings also reported that betalains degraded quickly at room temperature compared to −20 °C. In contrast, the temperature affected phenolic acids and antioxidative activity less.

García-Cayuela et al. [36] also extracted the same compounds of phenolic acids, flavonoids, and betalains from the whole fruit, pulp, and peel of six Mexican and Spanish prickly pear (*Opuntia ficus-indica* L. Mill.) cultivars. The highest concentration of betacyanins (1372–2176 μg/g dry whole fruit) was obtained from the purple cultivars of Spanish Morada and Mexican Pelota, whereas the highest amount of betaxanthin (435–488 μg/g dry whole fruit) was found in red Spanish Sanguinos and yellow Mexican Diamante cultivars. Additionally, the Spanish Morada cultivar has also been reported to have the greatest phenolic content of 49,012 μg/g dry peel.

Recently, Lončarić et al. [37] extracted phenolic, anthocyanins, and flavonol from blueberry pomace using green extraction methods such as high voltage electrical discharges (HVED), pulsed electric field (PEF), and ultrasound-assisted extraction (UAE). Antioxidant activity (0.83 mmol TE/g dw) and total polyphenols content (TPC) (10.52 mg of gallic acid equivalent (GAE) per g of dry weight (dw)) were both maximized through PEF-assisted extraction in the ethanol-based solvent. Meanwhile, PEF-assisted extraction produced the highest yields of anthocyanin (1757.32 g/g of dw) and flavanol (297.86 g/g of dw) in the methanol-based solvent. The highest yields of phenolic acid (625.47 g/g of dw) and flavonol (157.54 g/g of dw) were obtained in the ethanol-based solvent. These outcomes indicated PEF as a promising green extraction method that can increase the polyphenol extraction yield from blueberry pomace. Gulsunoglu et al. [38], on the other hand, extracted phenolic and flavonoid contents and further enhanced the compounds via fermentation with *Aspergillus* spp. The highest levels of total phenolics (1440 ± 37 and 1202 ± 88 mg GAE/100 g dm) and flavonoid contents (382 ± 47 and 495 ± 19 mg CE/100 g dm) were obtained from *A. niger* ZDM2 and *A. tubingensis* ZDM1, respectively. The antioxidant activity of apple peel fermented with these species was 3 and 5 times higher, respectively, than that of the unfermented sample, as measured by the CUPRAC and DPPH methods.

### 2.2. Vegetable 

A considerable amount of waste is generated throughout the cultivation, marketing, and processing of vegetables. This waste is particularly noticeable in the production of various vegetable products such as canned vegetables [39], vegetable juice [40], vegetable concentrate [41], vegetable jam [42], and vegetable-based fermented drinks [43]. As with fruit, the characteristics of vegetable products vary depending on the type of vegetable utilized. Moreover, vegetables can be grouped into categories based on the part of the plant that is used for food preparation. Some plants can even be classified in multiple categories since they have more than one edible part, such as beetroots, which can be consumed as both roots and leaves.

In a study by Lucera et al. [44], vegetable waste from broccoli and artichokes was utilized as a source of phenolic acids and flavonoids. These compounds were extracted and used to fortify cheese samples, resulting in a significant increase in antioxidant capacity. Another study by Salas-Millán et al. [45] explored the potential of broccoli stalk waste as a novel fermented health food using its own microbiota. The natural fermentation process was carried out at 25 °C for 6 days, followed by 6 days of storage at 4 °C. The highest levels of phenolic acids and flavonoids were observed on the third day of fermentation. Chitrakar et al. [46] extracted compounds from asparagus waste and turned them into powder to assess their impact on chip properties. Their findings indicated that the inclusion of asparagus waste led to a significant increase in total chlorophyll (161%) and total phenolic (46%) and total flavonoid (79%) contents. Meanwhile, Zhang et al. [47] isolated compounds from *Asparagus Officinalis* roots sourced from Chinese and New Zealand cultivars.

In a recent study by Sagar et al. [48], the flavonoid and total phenolic content of onion skin from 15 Indian cultivars were quantified. The results showed that the cv. “NHRDF Red” cultivar had the highest total phenolic and flavonoid contents, while cv. “Bhima Shubhra” had the lowest, demonstrating maximum and minimum antioxidant capacity for cv. “NHRDF Red” and cv. “Bhima Shubhra,” respectively. The study also concluded that the onion skin of cv. “Hissar-2” and cv. “NHRDF Red” were the best sources of flavonoids and natural antioxidants. Similarly, Milea et al. [49] utilized yellow onion waste to create ingredients with multifunctional activities. They obtained the extract through green solvent aqueous extraction of flavonoids, resulting in a total flavonoid content of 50.21 ± 0.09 mg quercetin equivalent (QE)/g dry weight (DW) and antioxidant activity of 250.81 ± 6.76 mM Trolox/g DW. Additionally, Joly et al. [50] extracted phenolic and flavonoid compounds from potato waste using maceration and heating-assisted extraction. The quantitative growth of these compounds was facilitated by heating-assisted extraction. The study also found that under the specified operation conditions, the total phenolic content of unpeeled potato samples was higher than that of peeled samples, indicating that the total phenolic content accumulated in the skin tissue.

In addition, Vaz et al. [51] investigated the waste of artichoke, red pepper, carrot, and cucumber to obtain concentrates of phenolic acids, flavonoids, and other dietary fibers. Their results showed that artichoke waste had the highest concentration of phenolic compounds at 8340.7 mg/kg, while red pepper, carrot, and cucumber had much lower concentrations at 304.4 mg/kg, 217.4 mg/kg, and 195.7 mg/kg, respectively. In another study, phenolic, flavonoid, anthocyanin, and carotenoid components were extracted from potato peel and flesh from four potato varieties. The concentrations of total phenolic, flavonoids, and anthocyanins were 1.58, 2.91, and 1.46 times higher, respectively, in the peel compared to the flesh. Antioxidant activity measurements using FRAP, DPPH, and ABTS assays were also 1.64, 2.19, and 1.66 times higher in the peel than in the flesh, respectively [52]. Moreover, Doulabi et al. [53] extracted phenolic, flavonoid, and anthocyanin compounds from eggplant peel waste using microwave-assisted extraction. They found that a higher microwave power, low ethanol concentrations, and liquid–solid ratios resulted in a greater yield of bioactive compounds and increased the total anthocyanin, phenolic, and flavonoid contents.

**Table 1:** Bioactive compounds from fruit and vegetable waste.

Fruits and Vegetable Waste	Bioactive Compounds	Extraction Method	References
Fruits waste			
Blackberry pomace	Phenolic acids, anthocyanins	Pressurized liquid extraction (PLE), supercritical fluid extraction (SFE)	[23]
Blueberry (*Vaccinium ashei*) juice		Hot water bath and steam pretreatments	[24]
Blackberry, blueberry and jaboticaba skin		-	[25]
Apple peels		-	[26]
Strawberry fruit peel		-	[27]
Raspberry pomace		Solid phase extraction (SPE)	[28]
Red dragon fruit peels		Methanol extraction	[29]
Tomato skin	Carotenoids, phenolic acids, and flavonoids	Supercritical CO_2_ extraction (SC-CO_2_)	[30]
Pumpkin seeds, peel, flesh		Aqueous phase extraction	[31]
Papaya peel		Ultrasound assisted extraction (UAE)	[32]
Melon peel		-	[33]
Red beetroot peel extract	Phenolic acids, flavonoids, betalains	Maceration technique	[34]
Red beetroot juice		Ultrasound assisted extraction (UAE)	[35]
Pulp and peel of prickly pear (*Opuntia ficus-indica* L. Mill) tissues		Single extraction	[36]
Blueberry pomace extract	Phenolic acids, anthocyanins, flavonol	High voltage electrical discharges (HVED), pulsed electric field (PEF), ultrasound-assisted extraction (UAE)	[37]
Apple peels	Phenolic, flavonoids	-	[38]
Vegetable by-products			
Broccoli and artichokes waste	Phenolic, flavonoids	Methanolic extraction	[44]
Broccoli waste (stalk)		-	[45]
Asparagus leaf waste		-	[46]
*Asparagus Officinalis* roots		-	[47]
Onion skin		Pressurized hot water extraction	[48]
Yellow onion skin		Hot water extraction	[49]
Potato waste		Maceration, hot water extraction	[50]
Artichoke, red pepper, carrot, and cucumber waste		Ultrasonic processor	[51]
Potato peel	Phenolic, flavonoids, anthocyanins, carotenoids	-	[52]
Eggplant peel	Phenolic, flavonoids, anthocyanins	Microwave-assisted extraction (MAE)	[53]

## 3. Extraction Methods

Many bioactive compounds can be found in fruit and vegetable waste. To separate, identify, characterize, and extract these compounds in the proper way, it is crucial to know where they come from and what methods can be used for a particular plant matrix. Extraction technique is necessary to extract the desired bioactive component from a complex plant matrix, to enhance the sensitivity and selectivity of the analytical approach, to change the bioactive compound into an identification and separation form, and to have reproducible extraction methods. The bioactive compound can be extracted from fruit and vegetable waste using conventional and nonconventional techniques [54]. Maceration and hydrodistillation, microwave-assisted extraction, supercritical fluid extraction, pressurized liquid extraction, and pulsed electric field are examples of extraction techniques of bioactive compounds.

### 3.1. Maceration and Hydrodistillation

This method is one of the extraction techniques used for the recovery of bioactive compounds. Alrugaibah et al. [55] observed the efficacy of natural deep eutectic solvents (NDES) in extracting procyanidins and anthocyanins from cranberry pomace with the assistance of ultrasounds. The study demonstrates that the NDES of a certain composition have greater extraction efficiency and more selectivity for procyanidins or anthocyanins from cranberry pomace than 75% ethanol. The NDES 2 with choline chloride:betaine hydrochloride:levulinic acid (1:1:2) and 32 mL water/100 mL NDES extracted the maximum concentration of procyanidins (32.5 mg/g). The most anthocyanins were extracted from NDES 8, which was made up of glucose:lactic acid (1:5) and 20 mL/100 mL water. The amount extracted was 1.58 mg/g, which was 1.79-fold as much as the yield from 75%. The greatest extraction yields predicted by artificial neural networking (ANN) and response surface methodology (RSM) were comparable to the experimental yields, indicating that artificial neural networks are an alternative or superior predictive modeling method to RSM. Wu et al. [56] extracted essential oils from grape and pomelo peel using hydrodistillation combined with electrofluidic pretreatment. Using a variety of approaches, the authors reported a 43% increase in the essential oil recovery yield from grape peel and a 93% increase from pomelo peel. Due to the breakdown of cell contents, fluid impedance decreased during the IEF pretreatment phase, and essential oil yield decreased slightly as frequency increased. Askin [57] compared the aroma profiles of essential oils extracted using hydrodistillation from orange peel waste. Some components—such as 1-nonanol, β-citral, E-2-decanol, and o-hydroxybiphenyl—which were negligible in oils obtained from fresh peels, were found to be significantly elevated in oils obtained from MW400.

Ramli et al. [58] extracted the *Citrus hystrix* (Kaffir lime) and *Ananas comosus* (pineapple) peels using maceration techniques. Compounds such as citronellal, linalool, citronellol, terpinen-4-ol, isoPulegol, and -cubebene were found during phytochemical screening of *C. hystrix* peels. Meanwhile, benzene, 1,3-bis (phenoxyphenoxy), 2-furancarboxaldehyde, 5-methyl-, 2-furancarboxal-dehyde, 5-(hydroxymethyl)-, and 4H-pyran-4-one, 2,3-dihydro-3, 5-dihydroxy-6-methyl were identified in *A. comosus* peel. Cvetanović et al. [59] also optimized the maceration extraction of tannins from *Tamarindus indica* L. seed. RSM revealed the optimal extraction parameters to be a methanol concentration of 69.99 percent, an extraction temperature of 23.38 °C, and a solvent-to-sample ratio of 1:20. Using the HPLC-ESI-MS/MS technology, the extract obtained under optimal circumstances was chemically characterized. Coelho et al. [60] extracted bioactive compounds from mango peel liqueurs (*Mangifera indica* L.) using the maceration technique. Maceration with pectinase produced liqueurs with a greater concentration of quercetin-3-O-glucopyranoside. In regard to mango varieties, the bioactive content of Haden liqueurs was higher than that of Tommy Atkins liqueurs. The liqueurs had a high level of antioxidant activity.

### 3.2. Microwave-Assisted Extraction

Microwave-assisted extraction (MAE) is a technology that utilizes microwave energy to extract phytochemicals. Carbone et al. [61] extracted kiwi juice pomace for the recovery of natural antioxidants through microwave-assisted extraction (MAE). On the extraction yield of total polyphenols, temperature (T) was the most impactful factor, followed by solid-to-solvent ratio € and solvent compositi€ (C), although extraction€me (E) did not have a significant linear effect (TPs). The total polyphenols (TPs) yield varied from 1.30–4.87 mg GAE g^−1^ dw. Doulabi et al. [53] also evaluated the extraction of bioactive compounds from eggplant peel waste by using microwave-assisted extraction methods. The extraction yield, TAC, TPC, and TFC rose significantly (*p* < 0.01) in parallel with the microwave power increase and the usage of lower ethanol concentrations and lower liquid–solid ratios. The maximum predicted extraction yield (3.27%), TPC (1049.84 µg GAE/mL), TFC (130.40 µg QE/mL), and TAC (6.99 mg/L), as well as the minimum predicted IC50 (0.52 mL/mg), were obtained simultaneously when power, extraction time, liquid–solid ratio, ethanol concentration, and pH of solvent were 269.82 W, 7.98 min, 5.01 mL/g, 73.49%, and 3.06%, respectively. Overall, effective extraction of bioactive components from eggplant peels was possible under optimal microwave settings. Kurtulbaş et al. [62] used the MAE method to extract bioactive components from peach waste. Based on the results obtained, the highest yields were obtained with an 80% ethanol solvent system at 500 W microwave power for 90 s. Under deep freezer (−20 °C) conditions, the total phenols, anthocyanins, and main phenolic components (p-hydroxybenzoic acid and p-coumaric acid) had calculated shelf lives of 111, 107, 88, and 83 days, respectively.

Furthermore, da Rocha and Noreña [63] extracted bioactive compounds from grape pomace using the MAE and UAE methods. The results showed that for both methods of extraction, the amounts of total phenolic compounds and antioxidant activity measured by ABTS and DPPH increased over time, and the best extraction condition was a microwave at 1000 W for 10 min. However the amount of phenolic compounds and antioxidant activity were lower than when exhaustive extraction was done with acidified methanol solution. Lasunon et al. [64] studied the effect of the MAE method on bioactive compounds from tomato waste. For hydrophilic parts, the best extraction conditions were 180 W for 90 s and 450 W for 30 s, which gave the highest total phenolic compound content (280.10 mg GAE/100 g) and total flavonoid content (9832.52 mg CE/100 g DM). The condition of MAE which yielded the high bioactive compound would exhibit lower antioxidant activity. Araújo et al. [65] extracted the bioactive molecules from avocado seeds by using the MAE method. The optimized extracts made with acetone and ethanol had a high polyphenolic content (307.09 ± 14.16 and 254.40 ± 16.36 mg GAE/g extract) and high antioxidant activity as measured by DPPH (266.56 ± 2.76 and 221.69 ± 20.12 mg ET/g extract), ABTS (607.28 ± 4.71 and 516.34 ± 11.81 mg ET/g extract), and ORAC (475.55 ± 47.82). MAE is a green, energy-efficient, and quick way to extract bioactive components from avocado seeds without affecting their antioxidant activity. This makes it a good alternative to traditional protocols in the herbal industry and medicine.

### 3.3. Supercritical Fluid Extraction

Supercritical fluids are commonly used in business as an alternative to solvent extraction and are considered “environmentally friendly” due to the removal of harmful solvents from operations. In this regard, Pellicano et al. [30] optimized supercritical fluid extraction (SPE) to extract bioactive compounds from tomato skin waste. After 80 min at a pressure of 550 bar, the greatest oil output of 79% was obtained. This oleoresin was determined to be the richest in carotenoids, with lycopene and β-carotene concentrations of 0.86 and 1.5 mg/100 g, respectively. Naringenin was the most abundant flavonoid found, with a maximum level of 84.04 mg/kg DW in oleoresin extracted at 550 bar, followed by caffeic acid (26.60 mg/kg DW). Kupnik et al. [66] extracted bioactive constituents from pomegranate peel waste by using a supercritical fluid extraction method. The LC-MS/MS approach was used to identify and quantify phenolic compounds in selected extracts. The contents of various flavonoids and phenolic acids were determined. At 20 MPa, SFE extract had the highest total polyphenol concentration (11,561.84 µg/g), with ellagic acid (7492.53 µg/g) being the most abundant. Madhumeena et al. [67] investigated the SPE method for the extraction of bioactive components from pineapple waste. The ferulic acid was then quantified using high performance liquid chromatography (HPLC). Ferulic acid is a hydroxycinnamic acid that can be transformed into vanillic acid (flavor compound). Ferulic acid was discovered to be present in 0.76968/100 g of pineapple peel powder.

Soldan et al. [68] extracted oleoresin from Capsicum annuum industrial waste using a supercritical fluid extraction method. Result shows that the total mass yields obtained by SFE varied between 9.38 and 10.08 %. The extracts contained bioactive substances, such as phenolics, flavonoids, fatty acids, and carotenoids, but possessed insufficient antioxidant activity. De Andrade Lima et al. [69] also used SFE to extract carotenoids from vegetable waste. From the results found in the research, the total carotenoid recovery (TCR) was greater than 90% *w*/*w* for most samples, and the most highly extracted compound was β-carotene (TCRs 88–100% *w*/*w*). According to the findings, the optimized SFE conditions can be utilized as a universal model for carotenoid extraction from various fruit and vegetable matrices, as well as a viable strategy for adding value to these waste streams by producing carotenoid-rich extracts. Romano et al. [70] extracted bioactive compounds from citrus peel using a supercritical CO_2_ method. When 20% ethanol was utilized as a cosolvent in both liquid (at 20 MPa and 20 °C) and supercritical (at 30 MPa and 60 °C) CO_2_ extraction, the best yields were obtained. Furthermore, the extracts prepared with liquid CO_2_ + 20% ethanol contained the most naringin (35.26, 44.05, and 19.86 mg g^−1^ in orange, tangerine, and lemon peel extracts, respectively) and terpenes, particularly limonene. These results indicate that extraction with a liquid CO_2_ and ethanol mixture could be a viable alternative to standard solvent extraction, using 80% less organic solvent and yielding extracts with good antiradical capacity and high volatile organic compound content.

### 3.4. Pressurized Liquid Extraction

Pressurized liquid extraction (PLE) is a method of solid–liquid extraction that uses pressurized liquids as the extractant solvent at elevated temperatures and pressures below their critical point. The PLE applies high pressures to the solvent, causing it to remain liquid past its typical boiling point [71]. Garcia et al. [72] investigated the recovery of bioactive compounds from pomegranate (*Punica granatum* L.) peel using pressurized liquid extraction (PLE). Based on the result obtained by the researchers, the TPC and punicalagin contents of PPE-PLE were 164.3 ± 10.7 mg GAE/g DW and 17 ± 3.6 mg/g DW, respectively. PPE-PLE demonstrated promising results in terms of AMA and cytotoxicity. Indeed, the AMA was impacted by the polyphenol content and other extracted bioactive components. Santos et al. [23] used feijoa (*Acca sellowiana (O. Berg) Burret*) peel to extract bioactive compounds via low- and high-pressure techniques. In this study, PLE proved to be the best extraction method for phenolics and antioxidants. Bioactive compounds such as ferulic, gallic and ellagic acid were identified using liquid chromatography–mass spectrometry (LC-ESI-MS/MS) as the major phenolic compounds found in feijoa peel extracts. Lasta et al. [73] applied the PLE technique for the recovery of phenolic compounds from beetroot waste. This study found that some of the abundant phenolic compounds, such as ferulic acid, vitexin, and sinapaldehyde, were obtained from the extracts. These findings suggest that the PLE technique may be a viable option for extracting phenolic compounds from beets that have a high biological potential and might be used in formulations for the nutraceutical, functional food, or pharmaceutical industries.

Andrade et al. [74] used ultrasound-assisted pressurized liquid method to extract anthocyanins from *Aronia melanocarpa* pomace. The extraction is performed in continuous mode to enhance the yields and the extraction yield was found to be increased by 19%. In a 200 W ultrasonic bath, optimal conditions of 70 °C, 180 bar, and a solvent content of 1.5% wt. citric acid was attained. These settings resulted in anthocyanin extraction of 88.0% by weight in 45 min. Pukalskiene et al. [75] recovered bioactive compounds from strawberry (*Fragaria x ananassa*) pomace using pressurized liquid extraction. The most prevalent elements in strawberry pomace extracts were quercetin-3-glucuronide; kaempferol-3-glucuronide; and tiliroside, ellagic, malic, succinic, citric, and p-coumaric acids. For the first time, the cytotoxicity, antiproliferative, and cellular antioxidant activities of strawberry pomace extracts obtained by PLE were reported. The PLE-EtOH extract demonstrated the greatest antiproliferative efficacy with no cytotoxicity. Both PLE extracts had a high antioxidant capacity and protected Caco-2 cells from stress. It is reasonable to believe that the biological activities of the extracts are related to the presence of the flavonoids (anthocyanins, catechins) and phenolic acids discovered and quantified in this study (ellagic, coumaric). Guzmán-Lorite et al. [76] compared pressurized liquids with high intensity focused ultrasounds for the extraction of proteins from pomegranate seed waste. Based on the findings, pressurized liquid extraction methods gained higher levels of proteins, peptides, and phenolics. Other interesting chemicals were discovered in addition to phenolic compounds, which were strongly coextracted via PLE using proteins such as azelaic acid.

### 3.5. Pulsed Electric Field

Pulse electric field (PEF) extraction is a nonthermal, green, and selective extraction technology that does not require a large amount of energy and that has been shown to be effective in short extraction times [77]. PEF has mostly been used in the food sector to reduce microbial growth/pasteurization and to improve the extraction of phytochemicals from fruit and vegetable industry waste [78]. Lakka et al. [79] evaluated pulsed electric field polyphenol extraction from *Vitis vinifera*, *Sideritis scardica*, and *Crocus sativus*. The finding shows that PEF treatment enhanced the recovery in total polyphenols for all the three plants examined. A considerable increase was observed in each plant examined and PEF condition used, albeit lower electric field intensities up to 1.4 kV/cm proved to be more beneficial. Total polyphenol content increased by 49.15%, 35.25%, and 44.36%, respectively, at the optimum electric field strengths of 1.4 kV/cm for *V. vinifera* and 1.2 kV/cm for *S. scardica* and *C. sativus*. Tehrani et al. [80] extracted valuable compounds in onion (*Allium cepa* L.) waste using a pulsed electric field (PEF). TPC, DPPH, FRAP, quercetin, and extraction yield were 48.912 ± 6 mg/kg, 50.366 ± 1%, 465.414 ± 5 µmFe_2_/l, 31.761 ± 0.5 mg/100 g, and 88.107 ± 1% respectively. The findings suggested that PEF could be a highly successful approach for continuous extraction of natural chemicals. Hwang et al. [81] also used *Citrus unshiu* peel waste for the recovery of hesperidin and narirutin using PEF method. Hesperidin concentration peaked at 46.96 ± 3.37 mg/g peel (dry basis) after PEF treatment at 120 s combined with SWE at 150 °C for 15 min, while narirutin concentration peaked at 8.76 ± 0.83 mg/g after PEF treatment at 120 s along with SWE at 190 °C for 5 min. Both hesperidin and narirutin concentrations increased with PEF treatment time. The PEF improved the extraction of hesperidin and narirutin by 22.1% and 33.6%, respectively. This work shows that PEF pretreatment can improve the SWE of flavonoids from *C. unshiu* peel.

Andreou et al. [82] applied pulsed electric field technique to enhance the product yield and waste valorisation in industrial tomato processing. According to the results obtained, The PEF treatment reduced peel detachment work by 72.3% without affecting ultimate tomato firmness. After PEF processing, the highest amount of tomato juice that could be made was 20.5% more than from an untreated sample. PEF at 5 kV/cm enhanced carotenoid extraction by 56.4% (14.31 mg lycopene/100 g tomato waste). The extraction of phenolic compounds from tomato waste increased (56.16 mg GA/kg) at PEF conditions of 2 kV/cm and 700 pulses. Lakka et al. [83] enhanced the recovery of polyphenols from *Rosa canina*, *Calendula officinalis*, and *Castanea sativa* using PEF method. The PEF approach appeared effective in enhancing polyphenol extraction from all three plant materials examined. In the case of *Rosa canina* fruits, the application of a moderate to high electric field, up to 1.4 kV/cm, boosted total and individual polyphenol recovery to 63.79% and 84%, respectively. Extraction of high-added-value compounds via PEF from olive leaves has been performed by Pappas et al. [84]. Using a pulse duration of 10 μs, the greatest PEF effect was recorded for aqueous ethanol at a concentration of 25% *v*/*v*. The total polyphenols increased by 31.85%, whereas the particular metabolites increased by 265.67%. The recovery of polyphenols was discovered to be dependent on the solvent, the duration of the treatment pulse, and the structure of the extracted metabolites.

### 3.6. Ultrasound Assisted Extraction

Ultrasound-assisted extraction (UAE) is a useful method of extraction that works better than traditional extraction. Some of the benefits of UAE are that it takes less time, is easier to use, uses less solvent, keeps temperatures down, saves energy, and increases yield. UAE has the chance to improve extraction yields through cavitation and better mass transfer [85]. Pectin is found in the cell wall and middle lamella of many plants, including fruits and vegetables. This has led many researchers to try to get pectin from waste peel, pomace, and rind of plants. Fruit peels, such as passion fruit [86], orange [87], banana [88], grapefruit [89,90,91,92], mango [93], and pomegranate [94]; vegetables, such as eggplant [95] and jackfruit [96]; and other parts, such as grape pomace [97], tomato waste [98], durian rind [99], and sunflower head [100] have been used to extract pectin. Pomegranate peel, grape pomace, tomato waste, grapefruit peel, orange peel, and eggplant peel have all produced pectin yields in excess of 25% when processed using UAE. Ultrasonic factors including frequency, power, duty cycle, power intensity, sonication duration, and extraction variables like temperature, LSR (liquid (solvent) to solid ratio), and pH of solvent all have a role in determining the final pectin yield either alone or via interactive effects. Several results have proven that UAE can boost pectin output and shorten the time required for extraction. Wang et al. [101] compared chemical extraction with UAE extraction of pectin from grapefruit peel and found that UAE extraction produced a greater yield (16.34%) and reduced extraction time by 37.7%.

Guandalini et al. [102] did the UAE of pectin from the residue of phenolic compound extracted mango peel and found a 50% increase in yield (from 5.61 to 8.6). The yield of UAE pectin from rehydrated mango peel increased by 31% (from 6.2 to 8.1) compared to pectin extraction without ultrasound without affecting the quality of pectin. Similarly, Oliveira et al. [86] observed a 1.6-fold increase in the pectin yield via UAE of passion fruit peel over chemical extraction. Grassino et al. [98] found that conventional extraction of pectin at 60 °C gave the highest yield but that 15 min UAE produced higher-quality pectin. For extraction at 80 °C, the yield of pectin was the same after 24 h of traditional extraction and 15 min of UAE. This shows very clearly that UAE speeds up the extraction process. Characterization of pectin extracted through the UAE has been performed to find out about its quality and properties. Eggplant peel, grape pomace, passion fruit peel, sunflower head, and grapefruit peel have all been used to obtain high-methoxyl pectin (degree of esterification >50%). A chosen frequency between 30 and 40 kHz has been used to extract anthocyanin out of grape waste [103], pectin out of tomato waste [98], hemicellulose out of grape pomace [104], and oil out of papaya seed [105]. The choice of a constant low frequency may have to do with the idea that fewer cavitation bubbles with larger diameters favor a large cavitation effect, which decreases as the frequency of the ultrasound keeps increasing [106].

Brahmi et al. [107] optimized the conditions for ultrasound-assisted extraction of phenolic compounds from *Opuntia ficus-indica* [L.] Mill. flowers. From the study, UAE was statistically more effective than traditional extraction (maceration and Soxhlet extraction) methods at removing bioactive chemicals. UAE extracted 24.4 ± 0.82 mg of total phenolics as mg gallic acid equivalent (GAE)/g of dry weight (DW). The UAE procedure significantly increased the yield and decreased the extraction time compared to conventional methods (maceration and Soxhlet extraction). González-Silva et al. [108] extracted phenolic compounds from *Psidium cattleianum* leaves using the UAE method, and their results were optimized using response surface methodology (RSM) by assessing the effect of time of extraction (X_ET_ = 2, 4, and 6 min), sonication amplitude (X_SA_ = 60, 80, and 100%), and pulse cycle (X_PC_ = 0.4, 0.7, and 1 s). According to the RSM, the optimal parameters for extracting the maximum soluble polyphenol content and yield from UAE (158.18 mg/g dry matter [DM] and 15.81%) comprise a sonication amplitude of 100% for four minutes at a pulse cycle of 0.6 s. Using the response surface approach, the conditions for ultrasonic aided extraction (UAE) of phenolic chemicals from rye bran (RB) were optimized by Iftikhar et al. [109]. The best conditions for UAE were time: 29 min, temperature: 66 °C, and solid-to-solvent ratio: 1:45 (g/mL), yielding a total phenolic content of 245.74 mg GAE/100 g dry weight. UAE resulted in a greater yield of total phenolic and flavonoid components than conventional extraction (CE), but only a minor difference was observed in the total flavonol and proanthocyanidin contents. In every antioxidant test, the UAE extract had more antioxidant activity (DPPH, FRAP, ABTS, reducing power, and H_2_O_2_).

## 4. Valorization of Fruits and Vegetable Waste into Valuable Applications

In parallel with the food waste reduction strategies, recovery and revaluing of crop wastes have been actively conducted. The valorization of crop wastes are discussed in detail in the subsections below.

### 4.1. Antioxidant

The food sector is becoming increasingly influential in the outcome of novel biomaterials featuring antioxidant characteristics as it has the advantage of enhancing the shelf life of food products [110]. Compounds that are rich in polyphenols, such as phenolic acids and flavonoids, have been reported to have excellent antioxidant capacity [111], as those compounds can scavenge the reactive oxygen species (ROS) present in the food products [112]. Recently, Rangaraj et al. [113] developed an active gelatin film incorporated with date fruit syrup waste extract (DSWE) as an antioxidant additive for food storage studies. The active gelatin/DSWE blend films show a high release profile of the active phenolic compounds and higher antioxidant capacity in an aqueous food medium when compared to lipid-based food stimulants. Venturi et al. [114] used potato peels, which contain bioactive compounds and have higher antioxidant power to enhance the qualitative parameters of fresh-cut apples. During storage, a substantial anti-browning effect and a slowing of the softening of fruits were identified. The observed results indicate that potato extracts are suitable as antioxidant supplements for fresh-cut fruits, hence minimizing the usage of hazardous chemicals. Kurek et al. [112,115] created a chitosan and pectin film mixed with blackcurrant pomace powder to reduce the losses of food production, and this film is focused on food coating and wrappers. Based on the research, the antioxidant activity of the film was increased 30-fold. This film is suitable to be used as active or intelligent packaging films for antioxidant activity of fresh produce.

Kam et al. [116] utilized durian leaf extract in gelatin-based film for antioxidant activity enhancement of biodegradable film as active packaging. This research revealed that gelatin-based film with added 0.5% crude extract of durian leaf has 17.6 times higher DPPH scavenging activity than the negative control sample. Gelatin film with 0.5% durian leaf extract reduced the oxidation of palm oil at a rate that was three times lower. Deshmukh et al. [117] also employed guar gum/carboxymethyl cellulose incorporated with litchi shell waste extract (GCH/LSE) for antioxidant active packaging. According to the results obtained by the researchers, GCH/LSE 20% shows the highest antioxidant activity, which is 91.52%. Active GCH/LSE films are good at maintaining roasted peanuts’ oxidative stability. The GCH/LSE films were proven to be effective antioxidant packaging for foods with a high lipid content. Orqueda et al. [118] tested an active edible film using red chilto (*Solanum betaceum*) peel and seed to reduce the oxidation of salmon filets during storage. Based on the experiment conducted, samples collected from chilto peel had the highest concentration of phenolic compounds. Antioxidant pectin-based film can be found in *S. betaceum* fruit peel, and it successfully shows remarkable antioxidant properties that protect the salmon filets.

Ribeiro et al. [119] created pectin–phenolic antioxidant films from mango peels for food packaging applications. Result shows that film with aqueous or methanolic extracts provides higher antioxidant activity based on the inhibition of the DPPH radical. The extracts, on the other hand, increased elongation and the water vapor barrier and also provided films with a greater antioxidant capacity, making them promising materials as active food packaging/coating, particularly for food products susceptible to lipid oxidation, such as edible nuts, fruits, and breakfast cereals. Merino et al. [120] developed a highly antioxidant bioplastic film from avocado peels and seeds. The findings suggest that combining hydrolysis, plasticization, and pectin blending is critical for obtaining materials with competitive mechanical properties, biodegradability, excellent oxygen barrier properties, high antioxidant activity, optical clarity, and component migration suitable for food contact applications. The created materials, which stand out for their antioxidant activity, natural composition, and ecologically safe technique of creation, thereby constitute an appropriate and sustainable alternative to conventional nonbiodegradable plastic food packaging materials. A new active coating was developed using *Cucumis metuliferus* (CM) fruit-extract-loaded acetate cellulose coating for antioxidant active packaging [121]. To assess the total phenolic components and antioxidant activity of CM, the extraction procedure was initially optimized. Release studies of CM from the cellulose acetate layer to a fatty food simulant verified the antioxidant efficiency of bilayer packaging.

Gaikwad et al. [13] created a biocomposite film made of polyvinyl alcohol and apple pomace that possesses antioxidant qualities and may be used in active food packaging applications for the storage of soybean oil. They discovered that the inclusion of apple pomace into PVA films increased the overall phenolic content and the antioxidant capabilities of the films in delaying lipid oxidation. Meanwhile, Matta et al. [122] created an active edible film of methylcellulose (MC) containing extracts of green apple (*Granny Smith*) skin by incorporating ethanolic extracts of freeze-dried apple skin (EEFD) and aqueous extracts of apple skin (AES). The results indicate that including extract of green apple skin into MC films increases the films’ overall phenolic contents and antioxidant capabilities. The potential for developing these films into functional packaging materials for food to ensure quality and safety and broaden the shelf life of packaged foods is therefore significant, and it represents an attractive alternative to the conventional packaging materials in terms of food safety and shelf life extension. Similarly, Han and Song [123] conducted a study on the antioxidant properties of mandarin (*Citrus unshiu*) peel pectin films comprising sage (*Salvia officinalis*) leaf extract for environmentally and biodegradable food packaging in which the biopolymer films were made from pectin derived from mandarin peel, a waste of fruit processing. Antioxidant activity of the film increased as the amount of sage leaf extract increased, which indirectly proved the antioxidant properties of sage leaf extract.

Furthermore, Saberi et al. [14] utilized pea starch and guar gum (PSGG) to produce food packaging that was incorporated with natural antioxidants comprising of epigallocatechin gallate (EGCG), blueberry ash (BBA) fruit extract, macadamia (MAC) peel extract, and banana (BAN) peel extract. The findings showed that EGCG-PSGG films had the most antioxidant power, followed by BBA-PSGG, MAC-PSGG, and BAN-PSGG films, at all concentrations (0.75–3 mg mL^−1^) when measured using DPPH radical scavenging ability assay, cupric reducing antioxidant (CUPRAC), and ferric reducing activity power (FRAP). Inclusion of natural additives has been demonstrated to improve the moisture barrier of the films. Shahbazi [124] combined ethanolic red grape seed extract and *Ziziphora clinopodioides* essential oil into chitosan and gelatin films to enhance the antibacterial, antioxidant, physical, and mechanical properties of the chitosan (Ch) and gelatin (Ge) films. The two most important chemicals in the ZEO are carvacrol and thymol, which provide antibacterial and antioxidant properties for ZEO. In addition, mango peel extract (MPE) has revealed its robust free-radical-scavenging capabilities and was combined with fish gelatin films to improve the physical, barrier, mechanical, and antioxidant capabilities of the developed film [125].

Interestingly, Melo et al. [126] integrated three chemical fractions derived from mango kernels to create active films: mango kernel starch (MKS) as a matrix, mango kernel fat (MKF), and phenolic extract (MKPE). MKPE was added to give the films active qualities such as primarily antioxidant and UV absorber. The active films are especially appealing for use as bags, pouches, or coatings for oxidizable foods. Additionally, Moghadam et al. [127] created edible antioxidant films using mung bean protein and pomegranate peel. The current study effectively enhanced mung bean protein-based edible films with varying amounts of pomegranate peel as a natural bioactive chemical to develop an antibacterial and antioxidant packaging system. Pomegranate peel, a low-cost by-product of the food industry, has the potential to improve the bio-functional qualities of mung bean protein film. Rodsamran and Sothornvit [128] developed a pectin fraction from pineapple peel to serve as biopolymer films. As expected, TPC and antioxidant activity against DPPH and ABTS radicals in pectin films rose significantly when the PPS to water ratio increased in both stimulation media. The results demonstrated that the whole pineapple peel pectin extract solution (PPS) can be used as a natural plasticizer to produce pectin films with enhanced film properties, particularly in terms of the water vapor barrier and antioxidant properties, and that this research has the potential to become an effective active film or coating for food applications.

Additionally, Vargas-Torrico et al. [129] created gelatin/carboxymethylcellulose active films with Hass avocado peel extract as berry preservation packaging. According to their findings, avocado extract gave active films antifungal and antioxidant properties and altered the physicochemical characteristics of active films. Talón et al. [130] created edible films with a polyphenol from thyme extract (TE) as an antioxidant incorporated into chitosan and starch matrix. Inclusion of TE in the polymer matrix has been shown to improve the mechanical properties of the film as well as providing great antioxidant capacity. The findings also suggested that these antioxidant films may be used for coating applications to extend the shelf life of items susceptible to oxidative processes. Moreover, Urbina et al. [131] employed 100% apple-waste-derived multicomponent films as antioxidants in developing an active packaging. The researcher creates nano papers (NPs) with higher hydrophobicity and antioxidant activity from bacterial cellulose. PHA coatings with varying concentrations of apple extract (e) with antioxidant activity were also applied to the nano papers (NP/PHA-e). The integration of apple extract in the PHA coating supplied free radical scavenging capability to the films since the extracts retained their antioxidant potential after the films were processed. Kurek et al. [11] examined blueberry and red grape skin extract incorporated with chitosan (CS) and carboxymethyl cellulose (CMC) as an antioxidant film. The result showed that blueberry and red grape skin pomace extracts gives a great antioxidant activity. The summary of fruits and vegetables waste used as antioxidant in food shown in Table 2.

### 4.2. Antimicrobial

As a result of recent worldwide food-borne microbial outbreaks, researchers have been looking for novel approaches to preventing microbial growth in food without compromising its flavor, texture, or safety. Use of antimicrobial packaging is one strategy that gives consumers more peace of mind about the quality and security of their food. Therefore, the use of polymers or antimicrobial agents to create barrier-enhanced or active packaging materials is a promising strategy for preventing the growth and dissemination of microorganisms in food as summarize in Table 3. Recently, Ali et al. [16] employed pomegranate peel particles (PGP) mixed with starch as antimicrobial films which can also be used as food grade packaging material. PGP suppressed the development of both gram-positive (*S. aureus*) and gram-negative (*Salmonella*) bacteria. The research showed that created films displayed good antibacterial properties against both *S. aureus* and *Salmonella*. It was observed that as the concentration of PGP increased, so did the inhibition zone of the films against the targeted bacteria. PGP was significantly more effective against *S. aureus* than it was against *Salmonella*. Comparably, Hanani et al. [132] investigated fish gelatin films’ antibacterial characteristics as active packaging using pomegranate (*Punica granatum* L.) peel powder (PPP). Inclusion of PPP increased the physicochemical properties of the films as well as providing antibacterial capabilities. One of the most sensitive bacteria to the active film was determined to be *Staphylococcus aureus* (*S. aureus*), followed by *Listeria monocytogenes* (*L. monocytogenes*) as well as *Escherichia coli* (*E. coli*). For *S. aureus*, the most significant inhibitory zone (7.00 mm) was detected surrounding the film containing 5% PPP. These findings indicate that fish gelatin incorporating PPP has tremendous potential as a compelling film with antibacterial capabilities and can help maintain food quality and extend shelf life. Saleem and Saeed [133] studied the orange (*Citrus sinensis* L.), yellow lemon (*Citrus limonia Osbeck*), and banana (*Musa acuminata*) peel extracts as wide-range natural antimicrobial agents for agrowaste minimization. This study discovered that gram-negative bacteria are more sensitive to the extracts, with *Klebsiella pneumoniae* (gram-negative) bacteria exhibiting the highest sensitivity to the yellow lemon peel extract and the largest zone of inhibition.

Jodhani and Nataraj [134] utilized aloe vera gel and lemon peel extract to maintain the shelf life of banana (*Musa* spp.). The antifungal properties of lemon peel extract have been successfully tested on *Colletotrichum musae*, a decay-causing pathogen in bananas. The coating extended the shelf life of bananas up to 9 days without any disease incident. The combination effect on aloe vera and lemon peel essential oil effectively reduced quality losses of bananas without the use of chemical preservatives and hazardous fungicides. Meydanju et al. [135] developed a biodegradable film from lemon peel powder combined with xanthan gum (XG) and TiO_2_–Ag nanoparticles to inhibit the growth of *Escherichia coli* and *Staphylococcus aureus*. The synergistic effect of XG/TiO_2_-Ag and lemon peel powder has been reported to increase the growth of inhibition zone diameter. Additionally, Alparslan and Baygar [136] utilized orange (*Citrus sinensis* [L.] *Osbeck*) peel and combined it with chitosan films to monitor and evaluate the shelf life of deepwater pink shrimp. The antimicrobial properties of the combined film were tested on *Bacillus subtilis*, *Staphylococcus aureus*, *Escherichia coli*, *Pseudomonas aeruginosa*, and *Candida albicans*. The combination of chitosan film and OPEO (CH+OPEO) was effective in extending the shelf life of fresh shrimp to 15 days, whereas the only chitosan-coated (CH) group had a shelf life of 10 days, and the uncoated samples (control) only had a shelf life of 7 days.

Furthermore, Agdar et al. [137] evaluated the effectiveness of the salep gum coating containing orange peel essential oil in inhibiting the microbial growth on fish filets stored in refrigerated temperature for 16 days. The combined film prevented the growth of total aerobic mesophilic, coliforms, psychrophilic, and lactic acid bacteria. Additionally, the shelf life of fish filets was extended to 12 days when coated with salep gum containing 0.5% orange peel essential oil. Ramli et al. [138] observed the antibacterial activity of durian (*Durio zibethinus*) seed and peel extracts for effective shelf life enhancement of preserved meat. In terms of antibacterial characteristics, studies indicate that seeds have promising antimicrobial properties against *E. coli*, *P. vulgaris*, *S. marcescens,* and *S. aureus* and greater inhibitory zone size than peeling. Mayeli et al. [139] innovated a coating layer enriched with zein and sour orange peel extract to act as an antibacterial coating for refrigerated fish. The developed coating reduced the number of *Enterobacteriaceae*, *Pseudomonas* spp., and psychrotrophic bacteria during the storage period. The combined effect of the coating layers extended fish freshness by an average of 12 days. Shukor et al. [140] innovated antimicrobial packaging films using jackfruit peel waste for the shelf life of cherry tomatoes. The samples were tested for 7 days and the antimicrobial activity was done using *Escherichia coli* and *Staphylococcus aureus*. Based on the results gained, both *Escherichia coli* and *Staphylococcus aureus* were inhibited by films containing 10 wt% thymol. The application of these active films to actual food demonstrated that cherry tomato samples exhibited a considerable reduction in fungus growth.

**Table 3:** Usage of peel waste as antimicrobial in food applications.

Source of Waste	By-Product	Targeted Food Products	Extraction Method	Antimicrobial Effects	References
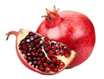 Pomegranate	Peel	-	Homogenization	Inhibited *S. aureus* and *Salmonella*	[16]
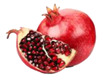 Pomegranate	Peel	-	Methanol extraction	Inhibited *Staphylococcus aureus, Listeria monocytogenes*, and *Escherichia coli*	[132]
Orange/Yellow lemon/Banana	Peel	-	Homogenization	Reduced *Klebsiella pneumoniae* (gram negative) bacteria	[133]
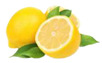 Lemon	Peel	Banana	Homogenization	Inhibited *Colletotrichum musae, Escherichia coli*, and *Staphylococcus aureus* Preserved the quality of the bananas Extended the shelf life of the bananas up to 9 days without any decay incident	[133,135]
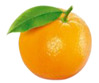 Orange	Peel	Deepwater pink shrimp, fish filets, fish	Methanolic and ethanolic extraction	Inhibited *Bacillus subtilis, Staphylococcus aureus, Escherichia coli, Pseudomonas aeruginosa, P. vulgaris, S. marcescens, S. aureus*, and *Candida albicans* Prevented the growth of total aerobic mesophilic, coliforms, psychrophilic, and lactic acid bacteria Extended the shelf life of fresh shrimp, fish filets, and fish	[136,137,139]
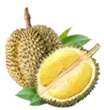 Durian	Peel	Meat	Rotary evaporation method	Inhibited *E. coli*, Enhanced the shelf life of preserved meat	[138]
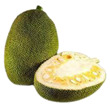 Jackfruit	Peel	Cherry tomato	Homogenization	Inhibited *E. coli* and *S. aureus* Extended the shelf life of cherry tomatoes	[140]

### 4.3. Antibrowning

The browning reaction, caused by an enzyme, is a serious issue with fresh produce. It alters the food’s quality in an unfavorable way, shortens its shelf life, and decreases its value on the market. Chemicals, especially sulfites, can inhibit enzymes responsible for browning. Reports indicate, however, that it poses health risks to consumers. Recently, Thivya et al. [141] discovered a new alternative to prevent the browning of fresh-cut apples and potatoes by proposing an active packaging composed of sodium alginate (SA) and carboxymethyl (CMC) along with shallot onion waste extract (SOWEs). Shallot onion waste extracts were reported to have an antibrowning effect due to their high content of polyphenols and antioxidant activity. The incorporation of SOWEs in SA/CMC active packaging has successfully inhibited the enzymatic reaction of polyphenol oxidase that is responsible for enzymatic browning in fruits and vegetables. Similarly, Tinello et al. [142] inactivated polyphenol oxidase enzyme using juices and distillates extracted from different onion varieties (white, yellow, and red) and inner layers of Borettana onion wastes. The inhibitory action of the extracted anti-browning agents was tested on commercial mushroom tyrosinase. The studies not only inhibited browning action but also extended the shelf life and improved the sensory properties of the food products. Interestingly, Liang et al. [143] successfully inhibited browning action in fresh-cut asparagus lettuce using extractable condensed tannins (ECTs) extracted from durian shells. The inhibitory action is predominant due to the presence of B-type procyanidins, propelargonidins and prodelphinidins, as well as a low degree of 3-O-galloylation, which are potent compounds that inhibit tyrosinase activity in fruits and vegetables.

Furthermore, Jirasuteeruk and Theerakulkait [144] investigated the anti-browning properties of mango peel extract from different varieties of mangoes. It was reported that mango extract from the Chok-Anan cultivar showed to have greater inhibition action compared to the Nam-Dok-Mai and Kaew cultivars. Chok-Anan mangoes exhibited high phenolics content and therefore inhibited polyphenol oxidase to a greater extent than the other two cultivars. Faiq and Theerakulkait [145] also reported that papaya peel crude extract has high bioactive phenolic compounds and hence has successfully inhibited browning reaction in potatoes, apples, and bananas. Martínez-Hernández et al. [146] produced an antibrowning agent from tomato skin which has a very high lycopene content. Fresh-cut apples were dipped in the lycopene microspheres, which successfully controlled the browning reaction after 9 days at 5 °C. Tinello et al. [147] utilized the juices from unripe grapes to serve as antibrowning and antioxidant agents to dried “Golden Delicious” apple slices. The appearance of the treated apple slices showed good results, demonstrating that the antibrowning agent of unripe grape juices effectively inhibited polyphenol oxidase activity. The usage of fruits and vegetables waste as antibrowning agents was summarized in Table 4.

### 4.4. Adsorbents

There is an alarming level of contamination in the aqueous medium, atmosphere, and geosphere due to the large number of toxic and poisonous chemicals released into our environment by various industries. Heavy metal pollution of the aqueous medium is a growing environmental crisis. Particularly problematic in treating industrial wastewater are toxic metals like zinc, mercury, lead, chromium, cadmium, nickel, and copper. Most of them cause cancer and are not biodegradable; they also form aggregates in living organisms. In order to safely remove pollutants and metallic elements from industrial effluents and wastewater, considerable in-depth industrial research has been conducted. As a result, several forms of fruit waste could be utilized to absorb or eliminate hazardous compounds, such as pesticides, heavy metals, and dyes.

Hussain et al. [148] study the flavonoids, total phenolics, and antioxidant properties of agricultural wastes and their capacity to eliminate pesticide waste using banana peels. Banana peels contain chlorophyll, hemicellulose, pectin, cellulose, and other low-molecular-weight compounds with higher antioxidant activity and phenolic content. At a concentration of 9 g, banana peels successfully decreased diazinon by 63.86%, while parathion was lowered by 50.34%. This study shows that agricultural wastes effectively remove diazinon from water, and their usage is environmentally friendly. Maia et al. [149] created activated carbon using banana peel waste using NaOH and pyrolysis to remove methylene blue. The results revealed that the activated carbon derived from banana peel waste has a vast surface area, which is required to remove blue methylene. The maximum dye removal efficiency was 99.8% when the MB starting concentration was 25 mg/L, the sorbent was 0.03 g, and the contact duration was 60 min. Banana-peel-activated carbon coated with Al_2_O_3_-Chitosan was developed. Ramutshatsha-Makhwedzha et al. [150] tested this study for the adsorption removal process of cadmium and lead from wastewater and found it to be successful. The BPAC composite was proven reusable until the third cycle of adsorption–desorption (% Re > 80). Based on the data obtained, the produced material could remove Cd^2+^ and Pb^2+^ up to 99.9% of the time.

Wang et al. [151] investigated activated carbon generated using tangerine seed waste for high-performance carbamate pesticide adsorption of plants and water. A novel activated carbon produced from waste tangerine seed was effectively synthesized in this work. Batch adsorption investigations revealed that the pseudo-second-order kinetic model and the Langmuir isotherm model predicted TSAC adsorption behavior well. Bendiocarb, metolcarb, isoprocarb, pirimicarb, carbaryl, and methiocarb have adsorption capacities of 7.97, 9.11, 13.95, 39.37, 44.64, and 93.46 mg/g, respectively. Furthermore, this adsorption mechanism was both spontaneous and exothermic. Gnanasekaran et al. [152] used orange peel extract with 3D ZnO/SnO_2_ for the removal of chlorophenol effluent. The research found that the existing various (Zn^2+^, Sn^4+^, and Sn^2+^) states aided in postponing the transmission of electron–hole recombination to achieve photocatalytic chlorophenol degradation. In this study, a green 3D composite system is used to absorb the chlorophenol by 77.5% through photocatalysis. Aminu and Oladepo [153] created orange-peel-mediated synthesis of silver nanoparticles for the detection of mercury (II) ions. Their results found that the golden-brown AgNPs colloid solution turned colorless when applied to water. AgNPs show good sensitivity and selectivity for the colorimetric detection of mercury (II) ions with the limit of detection og 1.24 × 10^−6^ mol/L.

Hassan et al. [154] also used date pits for the removal of organophosphorus pesticide from water. Three types of date pits were used as adsorbents, including roasted date pits, activated date pits, and nanoactivated date pits. The observation shows that nanoactivated date pits have high removal percentage and removal capacity of profenofos from aqueous solution compared to roasted date pits and activated date pits. Mohammad and El-Sayed [155] created activated carbon peach stones for the removal of imidacloprid. The researcher used two types of activated carbon (PSAC 300 and PSAC 500). The result shows that both PSAC 300 and PSAC 500 were able to remove imidacloprid, with about 80% and 99% removal efficiencies, respectively. Overall, PSAC 500 exhibited the maximum adsorption capacity of 39.37 mg/g. Al-Ghouti and Sweleh [156] employed activated carbon (ACOS) from black and green olive stones to remove methylene blue (MB) in water successfully. It was also discovered that black activated carbon olive stones had the most significant N%, H%, and C% prior to adsorption. Furthermore, the highest absorption of methylene blue occurred at the optimal pH value of 10. Methylene blue adsorption capacities were 714 mg g^−1^ and 769 mg g^−1^ for black and green activated carbon olive stones, respectively.

Batool et al. [157] removed organochlorine pesticides (OCPs) using a zerovalent iron (Fe^0^) supported on biochar nanocomposite (Fe^0^-BRtP) from *Nephelium lappaceum* (Rambutan) fruit peel waste. The experiment found that Fe^0^-BRtP combined the advantages of adsorption and dechlorination of OCPs in aqueous solution and up to 96–99% removal was obtained within 120 min. The removal efficiency of regenerated Fe^0^-BRtP was 89–92% after being reused five times. This Fe^0^-BRtP nanocomposite could be used as a green and low-cost prospective material for adsorption and reduction of OCPs in aquatic environments. Shakoor and Nasar [158] used citrus limetta peel waste as a methylene blue dye adsorbent. Langmuir adsorption isotherm was found to be the best fit for the data. The maximum adsorption capacity for a monolayer coverage was found to be 227.3 mg/g. The results show that CLP is a very effective, low-cost method of removing dyes from wastewater. Salama [159] investigated cellulose grafted with soy protein isolate (SPI) to remove organic dyes from wastewater. This method exhibited adsorption capacity up to 454 mg/g. The efficiency of MB removal was 95% after four adsorption-desorption cycles. This method is a new sustainable, cost-effective, and reusable hybrid material which can successfully adsorb dyes in wastewater. Goksu and Tanaydin [160] devised a technique for removing crystal violet (CV) color that uses almond shells as an effective adsorbent, and according to the findings of this study, almond shells are effective adsorbents for extracting crystal violet (CV) from aqueous solutions. On almond shells, the excellent adsorption capacity was reported to be 1.075 mg g^−1^. According to the results of the adsorption experiment, almond shells might be utilized to effectively remove CV in aqueous solutions (Table 5).

### 4.5. Indicator in Packaging

**Figure 1:**
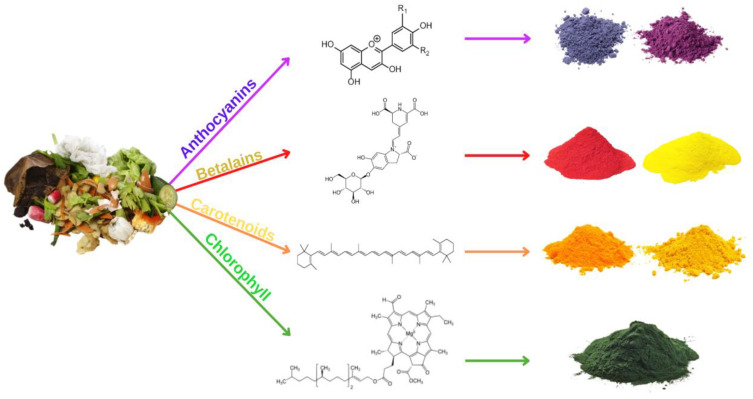
Natural source of pigments.

Petrochemical-derived synthetic pigments have been widely employed in a variety of food items. However, these colors negatively impact human health, making it necessary for the scientific community to search for safer, natural, and eco-friendly pigments. Recently, the pigments industry has expanded fast due to its numerous applications in food. As a result, it requires sustainable pigments manufacturing from renewable bio-resources. The valorization of vegetal wastes can fulfill the needs of natural pigment production at the industrial scale for food, medicinal, and cosmeceutical uses (Table 6). Natural colors, such as anthocyanins, betalains, carotenoids, and chlorophylls, are abundant in these wastes [161]. Figure 1 below shows the source of natural pigments.

#### 4.5.1. Anthocyanins

Anthocyanins are vacuolar polyphenolic pigments that are naturally occurring, water-soluble, and nontoxic [162]. There are roughly 700 different anthocyanin structures recognized, and they have been found in red cabbage [163], black carrot [164], red radish [165], and purple sweet potatoes [166] as well as in blackcurrants [167], cherries [168], and berries [169]. Anthocyanins are flavonoids, and the most trivial anthocyanidins found in the majority of colored plants include pelargonidin, delphinidin, cyanidin, petunidin, peonidin, and malvidin [170]. Anthocyanins differ in hue due to structural changes in hydroxyl groups, their quantity, the location of sugar moieties, cultivar type, and agricultural conditions [171].

Recently, Santos et al. [172] developed an active intelligent packaging based on sodium alginate films incorporated with 40% of *Clitoria ternatea* extract (CTE) to monitor the quality of milk, pork, and shrimp. The proposed packaging showed a colorimetric potential as CTE is a pH sensitive compound and it changes color at different pH. The blue color of the film changed to purple and green when loaded with 40% CTE and used to evaluate the perishability of milk and meat products, respectively (shrimp and pork). Zhao et al. [173] also utilized waste from purple sweet potato peel extracts (PPE) and combined it with sodium alginate to produce a pH-sensitive intelligent indicator film. The designed film changes color from pink to blue in the presence of total volatile nitrogen, indicating the freshness level of chicken stored at 4 °C for 5 days and 25 °C for 60 h. Similarly, Abdillah et al. [174] developed a halochromic indicator film composed of arrowroot starch/iota-carrageenan incorporated with anthocyanins extracted from Kyoho skin (KSE) to monitor the freshness of shrimp. Indicator films incorporating natural KSE anthocyanin showed pH sensitivity by changing color from purple to red in an acidic environment, from purple to green in an ammonium environment, and from purple to yellow in highly alkaline conditions.

Esfahani et al. [175] innovated an intelligent edible film from cassava starch and pomegranate peel powder (PPP) to monitor the freshness of lamb meat. The intelligent edible film exhibited color changes from red to green when tested on lamb meat stored at 25 °C due to the presence of total volatile basic nitrogen (TVB-N) in meat. Additionally, Capello et al. [176] extracted anthocyanins from agricultural waste of jabuticaba fruit (*Plinia cauliflora*) and purple sweet potato (*Ipomoea Batatas* L.) peels to serve as colorimetric indicator films. At the recommended storage temperatures, changes in color from red to blue were clearly evident in both colorimetric indicator films used to track meat freshness. He et al. [177] proposed pH-responsive color absorbent pads from polyvinyl alcohol (PVA), agarose (AG), and purple sweet potato anthocyanins (PSPA) to evaluate the freshness of meat. It was found that the pads’ colors changed noticeably as the pH was adjusted from 3 to 10, with the pad containing 9% PSPA exhibiting the most distinctive color change from pink to green. As a result, the agricultural waste has promising future applications as a colorimetric indicator for fresh meat, which would significantly contribute to the maintenance of food safety and the enhancement of food storage quality.

#### 4.5.2. Betalains

Regarding naturally occurring pigments, betalains are second only to anthocyanins and are primarily classified as betacyanins and betaxanthins [178]. These pigments give fruits and vegetable red-purple (betacyanin) and yellow-orange (betaxanthin) hues, respectively [179]. These chemicals are widely found as pigments in *Beta vulgaris* (beetroot) [180], *Opuntia* (prickly pears) [181], *Hylocereus* [182], and *Mammillaria* species [180]. Betalains are betalamic acid immonium conjugates containing amino groups and cyclic-dihydroxyphenylalanine (cyclo-DOPA) [183]. Betaxanthins are betalamic acid moieties synthesized with amino acids or amines. Condensation interactions between betalamic acid and cyclo-dopa produce betanidin, a betacyanin precursor. Betanidins have 29 distinct structures in nature due to glycosylation or acylation. Betanins have also been subjected to chemical reactions such as decarboxylation, deglycosylation, hydrolysis, isomerization, and dehydrogenation [184,185]. However, no published study specifies the number of betanin isomers or breakdown products.

Contemporarily, Ai et al. [186] developed a real-time intelligent film from film-forming substrates (FFS), glycerol, and red pitaya peel (RPP) extract to monitor the freshness level of pork. Betacyanins were extracted from red pitaya peel to act as a pH-sensitive indicator. In order to detect deterioration in the freshness of pork, the pH of the film was adjusted to 7.0. This film was more sensitive to ammonia than the original film, which had a pH of 4.3. During the deterioration process of pork, a change in film color was observed which correlated with the built-up total volatile base nitrogen. Kanatt [187] designed an active/intelligent packaging film constructed from polyvinyl alcohol (PVA) and gelatin incorporated with *Amaranthus* leaf extract (ALE) to evaluate the meat freshness of fish and chicken. Due to the rich bioactive compounds of ALE, the packaging provided antioxidants by minimizing oxidative rancidity, antibacterial by preventing microbial growth and intelligent properties to the film by showing evident color changes from red to yellow on the spoilage of the meat. Interestingly, the shelf life of the meat was extended up to 12 days.

Meanwhile, Ateteallah et al. [188] utilized beetroot juice containing betalains and carrot pulps as natural colorants to improve color and natural flavor and promote healthy constituents in the ice cream. Additionally, the addition of 5% of betalains to ice cream aids in improving total phenol and antioxidant properties of the ice cream. Hernández-Carranza et al. [189] utilized the peel and mucilage of red cactus pear to improve the color, total flavonoids, total phenolic compounds, total betalains, and antioxidant capacity of yogurt. The results reported that the betalains produce a nice magenta color in yogurt. Additionally, Fathordoobady et al. [182] utilized the peel of red dragon fruit (*Hylocereus polyrhizus* L.) and extracted betacyanin compound from it. Betacyanins were loaded into alginate microspheres and were characterized in terms of their encapsulation efficiency, particle size, uniformity, and morphology. Data demonstrated that alginate-loaded betacyanin extract can be a promising microcapsule that serves as a carrier for the delivery of antioxidants. Alginate-microsphere-loaded betacyanins have proven to be an efficient model for use as food colorant additives and for oral delivery.

#### 4.5.3. Carotenoids

Carotenoids are essential natural pigments which are lipophilic and account for the red, yellow, or orange color spectrum in various fruits and vegetables. Carotenoids are found in all trans and cis isomers [177,190,191]. Carotenes (-carotene, -carotene, and lycopene) and xanthophylls (lutein, zeaxanthin, and -cryptoxanthin) are the primary types of carotenoids [192]. Although both carotenes and xanthophylls include phytochemical chains of hydrogen and carbon, the presence of hydroxyl groups that indicate oxygenated carotenoids in structure distinguishes them [193].

Gungor and Torun [194] extracted β-carotene from peels of pumpkins using the maceration, microemulsion, and ultrasound-assisted methods with sunflower oil as the solvent. Data reported that mayonnaise made with sunflower oil rich in β-carotene was well received sensory-wise, and it retained all of its other distinctive qualities. The results of yellow color and peroxide values demonstrated that β-carotene fortified mayonnaise was more resistant to oxidation during storage than the control mayonnaise. Sharma et al. [195] employed leftovers of the internal fluffy portion along with fibrous strands of ripe pumpkin to extract β-carotene pigment. The extracted carotene pigment can then be used as a natural food colorant in processed food products, increasing both the visual appeal and nutritional value of the product due to the role of β-carotene as a precursor of vitamin A. Lombardelli et al. [196] developed a novel food colorant of carotenoid-containing chromoplasts from the unsold tomatoes. The stability of carotenoid-containing chromoplasts has been studied, and the chromoplasts have been reported to retain the colorimetric parameters of red-orange pigments when stored at 4 °C and 25 °C in the dark.

#### 4.5.4. Chlorophyll

Chlorophyll is a natural oil-soluble, amphiphilic green pigment found widely in plants [197]. Chlorophyll molecules have a lipophilic hydrocarbon tail and a hydrophilic (porphyrin) group head (phytol group) [198]. It is often considered insoluble in polar solutions because of its lipophilic hydrocarbon chains as a phytol tail [199]. Chlorophyll in plant meals is classified into chlorophyll a (blue-green) and chlorophyll b (yellow-green) [200]. Chlorophyll a has a methyl group (-CH3) at position 7-carbon, whereas chlorophyll b has an aldehyde group (-CHO) [201]. The demand for chlorophyll is constantly growing owing to increased knowledge of the usage of natural colorants and the health advantages they provide.

Aznury et al. [202] extracted chlorophyll from moringa leaf and incorporated it in yogurt, providing a green color. The higher the amount of moringa leaf extract, the darker the yogurt due to the high concentration of chlorophyll. Additionally, the addition of moringa leaf extract to yogurt samples has been reported to have a fairly high concentration of vitamin C and was highly preferred. Meanwhile, Rohajatien et al. [203] employed pegagan leaf (*Centella asiatica* L.) extract to improve the physical properties and antioxidant attributes of mochi ice cream. Addition of pegagan leaf extract produced yellowish-green mochi ice cream with an excellent antioxidant capacity. Molina et al. [204] extracted chlorophyll pigments from the aerial parts of carrot (*Daucus carota* L.) and tomato (*Solanum lycopersicum var. cerasiforme*). The findings reported that aerial parts of the tomato have higher chlorophyll concentration than carrot. The evaluated waste products showed promising results for use as sources of natural pigments with potential application in a variety of industrial fields as colorants, including the food, pharmaceutical, and cosmetic industries.

**Table 6:** Fruits and vegetables waste as natural colorant/indicators in food applications.

Sources of Waste	Natural Pigments	Extraction Method	Function	Application	Results	References
*Clitoria ternatea* extract (CTE)	Anthocyanin	Homogenization	Freshness indicator	Milk, shrimp, and pork	Changed color from blue to purple (milk) and blue to green (meat products)	[172]
Purple sweet potato peel extracts (PPE)	Anthocyanin	Homogenization	Freshness indicator	Chicken	Changed color from pink to blue in the presence of total volatile nitrogen	[173]
Kyoho skin extract	Anthocyanin	Homogenization	Freshness indicator	Shrimp	Changed color from purple to red in an acidic environment, from purple to green in an ammonium environment, and from purple to yellow in highly alkaline conditions.	[174]
Pomegranate peel powder	Anthocyanin	Homogenization	Freshness indicator	Lamb	Changed color from red to green in the presence of total volatile basic nitrogen (TVB-N)	[175]
Jabuticaba fruit (*Plinia cauliflora*) and purple sweet potato (*Ipomoea Batatas* L.) peels	Anthocyanin	Homogenization	Freshness indicator	Meat pieces	Changed color from red to blue as the pH increased	[176]
Purple sweet potato peel	Anthocyanin	Homogenization	Freshness indicator	Meat	Changed color from pink to green when pH changed from pH 3 to pH 10	[177]
Red pitaya peel	Betacyanins	Homogenization	Freshness indicator	Pork	Changed film color with the built-up total volatile base nitrogen.	[186]
Amaranthus leaf extract	Betalains	Homogenization	Freshness indicator	Fish and chicken	Prevented microbial growth Minimized oxidative rancidity Extended shelf-life to 12 days Changed color from red to yellow when the meat spoiled	[187]
Beetroot juice	Betalains	Homogenization	Natural food colorant	Ice cream	Improved the color and natural flavor Promoted healthy constituents Improved total phenol and antioxidant properties	[188]
Peel and mucilage of red cactus pear	Betalains	Hot water extraction	Natural food colorant	Yogurt	Improved the color, total flavonoids, total phenolic compounds, total betalains and antioxidant capacity of yogurt Provided a nice magenta color of yogurt	[189]
Peel of red dragon fruit (*Hylocereus polyrhizus* L.)	Betacyanins	Supercritical fluid extraction	Natural food colorant	-	Served as a promising microcapsules that serve as carriers for delivery of antioxidants	[182]
Peels of pumpkin	β-carotene	Maceration, ultrasound-assisted extraction	Natural food colorant and antioxidant	Mayonnaise	Provided yellow color to the mayonnaise Prevented oxidative rancidity Maintained the sensory qualities of mayonnaise	[194]
Leftovers of the internal fluffy portion along with fibrous strands of ripe pumpkin	β-carotene	Homogenization	Potential food colorant	-	Expected to increase both the visual appeal and nutritional value of the product due to the role of ß-carotene as a precursor of vitamin A	[195]
Unsold tomatoes	Carotenoid	Enzymatic-assisted extraction	Food colorant	-	Retained the colorimetric parameters of red-orange pigments when stored at 4 °C and 25 °C in the dark	[196]
Moringa leaf	Chlorophyll	Homogenization	Food colorant and functional food	Yogurt	Provided green color to the yogurt Improved the vitamin C content of the yogurt	[202]
Pegagan leaf extract	Chlorophyll	Homogenization	Food colorant and antioxidant	Mochi ice cream	Produced yellowish-green mochi ice cream Provided excellent antioxidant capacity.	[203]
Aerial parts of carrot (*Daucus carota* L.) and tomato (*Solanum lycopersicum var. cerasiforme*)	Chlorophyll	Maceration, ultrasound-assisted extraction	Potential colorant	-	Obtained high level of chlorophyll for future applicability studies	[204]

### 4.6. Enzymes

In the industrial sector, enzymes play a crucial role in reducing chemical loads, eliminating toxic substances, and reducing pollution [205]. Industrial enzymes currently have a market value of USD 5.9 billion, with a projected market value of USD 8.7 billion in 2026 and a compound annual growth rate of 6.5% [206]. A wide range of enzymes are commonly used in the industry, including lipases, carbohydrases, proteases, and polymerases [207]. Most of the waste produced by the food industry is lignocellulosic, which contains enzymes that are essential raw materials for production and processing [208]. As an example, bromelain, which is extracted from pineapple peel, can improve food digestion [209] and soften beef meat [210] and can be transformed into value-added products (Table 7).

Previously, Hussain et al. [211] extracted bromelain from pineapple core using the maceration extraction method. A maceration of chicken breast meat with 100% core extract shows an 86% reduction in hardness and pH decreases from 5.87 to 4.99. Meanwhile, Singh et al. [212] analyzed the effect of bromelain enzyme extracted from four different pineapple wastes—peel, stem, core, and crown—on tenderizing chicken and beef meat. Rizqiati et al. [213] investigated the effect of drying on cayenne pineapple crown enzyme characteristics—including protein content, activity units, and specific activity—in terms of moisture content, yield, and characteristics. The results obtained showed that the optimal drying temperature was 55 °C because it had the highest moisture content, protein content, and enzyme characteristics as well as the longest immersion duration for the best texture of meat. Lipases have been applied as food additives in the modification of taste in the food industry [214]. Recently, Okino-Delgado et al. [215] examined different varieties and fractions of orange wastes as sources of lipases. Among the fruit varieties studied, bagasse, peel, and frit lipases showed optimal pH values between 6.0 and 8.0 and optimal temperatures between 30 °C and 60 °C. Next, Tambun et al. [216] successfully produced fatty acids directly from avocado seeds by activating the lipase enzyme. The highest fatty acid content found was 11.67%. Furthermore, there is an enzyme called papain present in the bark, leaves, and fruit of the papaya plant [217].

Papain is extracted from fruit and stem latex can be utilized as a primary ingredient in brewing and winemaking [218]. Singla et al. [32] previously investigated the effects of microwave treatment and enzyme addition on ultrasound-assisted extraction (UAE) of bioactive compounds and antioxidant activity from papaya peels. Extracts prepared using UAE extraction were found to have antioxidant activity, indicating that they are useful for preparing plant extracts that contain antioxidants. Phothiwicha et al. [219] evaluated the bioactivity of papain extracted from Chaya and papaya stalks on beef meat. The texture and mastication of beef samples marinated with papain had a softer texture and were easier to chew after the marination. Amylases can therefore be used in a variety of industries, such as food, fermentation, and pharmaceuticals [220]. At present, Abdullah et al. [221] produced a safe sweetener in the form of glucose syrup using alpha amylase extracted from mango seed core, which can be used as an alternative to artificial sweeteners through enzymatic reactions. In a study which tested the amylase production ability of *Bacillus subtilis* K-18 (KX881940) by hydrolyzing potato peels as carbon sources, Mohtaq et al. [222] found that the bacterium can produce amylase as a result of starch hydrolysis.

**Table 7:** Application of enzyme extract from fruit and vegetable waste in the food industry.

Enzyme	Fruits and Vegetable Waste	Extraction Method	Application	References
Bromelain and protease	Core and seed of pineapple and jackfruit	Homogenization	Increasing the tenderness of tough muscle in bovine meat	[210]
Bromelain	Pineapple core	Homogenization	Reduction in hardness of chicken meat	[211]
	Peel, stem, core, and crown of pineapple	Deionized water	Tenderization of chicken and beef meats	[212]
	Pineapple crown	Homogenization	Improve meat texture	[213]
Lipase	Bagasse, peel, and frit lipases from the orange	Inline extractor	Juice production	[215]
	Avocado seed	Extract using n-hexane solvents	Production of fatty acids	[216]
Papain	Papaya peel	Ultrasound assisted extraction	Source of bioactive compounds for food	[32]
	Chaya and papaya stalks	Enzyme-assisted extraction	Halal beef tenderizer	[219]
α-amylase	Mango seed core	Homogenization	Glucose syrup alternative substitute	[220]
	Potato peel	Homogenization	Hydrolysis of starch to produce sugars	[222]

## 5. Current Status, Opportunities and Prospects for Fruits and Vegetables

Fruits and vegetables are agricultural commodities that generate waste during post-harvest processing, distribution, and consumption. While some by-products are utilized as fuel, construction materials, or animal feed, most of it is discarded. However, many agricultural by-products have been found to possess good nutritional properties and can be used as functional foods and components, thereby reducing production costs and improving food sustainability. Utilizing fruit and vegetable waste to boost the nutritional content of functional foods can also help address the impending food security crisis. Although there are safety concerns surrounding the use of these by-products for human consumption, thorough evaluation of factors such as the source of the by-products, processing methods, potential contaminants, nutritional composition, and health benefits or risks can ensure the safety of valorized by-products [223,224] Adhering to food safety regulations and conducting thorough testing and analysis can unlock the potential of fruit and vegetable by-products while ensuring consumer safety.

However, innovative fruit and vegetable waste is currently unavailable in the market, which is a significant gap in today’s sustainability-focused world. The high-risk investment involved in fruit and vegetable waste recycling is one possible reason for this gap. Additionally, consumers may perceive by-products as unsafe or unacceptable for consumption, leading to reluctance to purchase food containing them. To develop a better ecosystem for valorization of fruit and vegetable waste, a stronger technoeconomic perspective is required with a focus on low-volume, high-value, and better-sustainability alternatives that utilize green methods for extraction. Opportunities for fruits and vegetables waste have expanded in recent years, including nutraceuticals, flavoring agents, bioactive chemicals in packaging, and waste nanoparticles. These waste-derived value-added substances can be ingested in modest quantities, offering additional advantages. Thus, it is crucial to explore promising technologies for effective fruit and vegetable waste valorization in the near future.

## 6. Conclusions and Future Perspectives

In recent years, there has been a growing interest in utilizing waste and by-products generated from agriculture and food manufacturing. Fruit and vegetable waste, which includes peel, skin, fruits, kernels, seeds, and leaves, offers a plethora of benefits—such as antioxidant, antimicrobial, and antibrowning properties—as well as bioactive chemicals that can be utilized in various applications. This review focuses on the potential utilization of food waste for its antioxidant, antimicrobial, antibrowning, adsorbent, natural pigment, and enzyme properties. The extraction of natural pigments requires innovative and economically feasible resources, suitable extraction techniques, advancements in bioprocessing technologies, new inventions in pigment stabilization, applicability of a technique for a wide range of pigments, the possibility of developing pigment-enriched food ingredients and bioactives, and a trend towards developing pigment-enriched foods and bioactives. While green environmental techniques can absorb toxic compounds, such as heavy metals and pesticides, future research should focus on addressing concerns such as incomplete degradation of hazardous substances, high operational costs, high energy requirements, lower efficiency, and the regeneration of bioadsorbents for future treatments. It is important to note that before employing functional compounds derived from FVBP, additional research, such as toxicological tests, is required to ensure that the constituent is free of pesticides and other harmful chemicals. Bioactivity research is also necessary to determine the bioaccessibility and bioavailability of phytochemicals derived from these wastes.

## Figures and Tables

**Table 2:** Usage of fruits and vegetables waste as antioxidant in food applications.

Source of Waste	By-Product	Matrix	Extraction Method	Antioxidant Effects	References
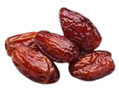 Date	Seed	Gelatin	Soxhlet extraction	Demonstrated good packaging properties with 25 wt% DSWE Exhibited better storage stability of canola oil Reduced peroxide (POV) and p-anisidine (PV) values Reduced values of canola oil samples Prevented lipid oxidation in canola oil.	[113]
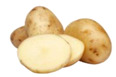 Potato	Peel	-	Solid/liquid extraction	Preserved the qualitative parameters of cut apples Provided anti-browning effect Slowed down softening of fruits	[114]
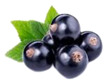 Blackcurrant	Pomace	Chitosan, pectin	Microwave assisted extraction (MAE), ultrasound assisted extraction (UAE), pulsed electric extraction (PEE), accelerated solvent extraction	Improved water permeability of the active film Increased antioxidant capacity to 30-fold Changed color after exposed with different pH buffers	[115]
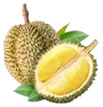 Durian	Leaf	Gelatin	Ultrasound-assisted extraction (UAE)	Exhibited higher DPPH scavenging activity of 17.6 with the presence of 0.5% leaf extract Reduced oxidation rate in palm oil Revealed no significant improvement in terms of water vapor permeability	[116]
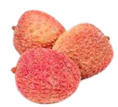 Lychee	Shell	Guar gum, carboxymethyl cellulose	Homogenization	Exhibited good antioxidant activity Maintained oxidative stability in roasted peanuts Reduced tensile strength in the presence of litchi shell extract Improved elongation at break in the presence of litchi shell extract Increased the UV–light barrier properties	[117]
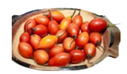 Red chilto	Seed, peel	Pectin	Homogenization	Extracted the pectin from the peel of *S. betaceum* fruits Incorporated pectin extract as film-forming matrix Extended shelf-life of salmon filets Reduced protein and lipid oxidation	[118]
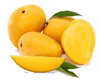 Mango	Peel	Pectin	Methanolic extraction	Showed high antioxidant capacity in terms of inhibition of DPPH radicals Inhibited the growth of Gram-positive and Gram-negative bacteria Reduced water permeability Showed good thermal stability	[119]
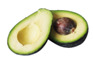 Avocado	Peel, seed	Pectin	Homogenization	Provided good mechanical properties of the antioxidant film Exhibited optical clarity, Provided excellent oxygen barrier properties Demonstrated high antioxidant activity	[120]
* 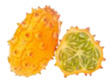 * *Cucumis metuliferus*	Extract	Cellulose acetate (CA), low-density polyethylene (LDPE) films	Aqueous extraction	Maintained high transparency of the film Improved oxygen barrier performance Reduced oxidation of fresh-cut apples	[121]
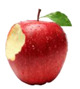 Apple	Pomace	Polyvinyl alcohol (PVA)	Homogenization	Improved total phenolic content Enhanced antioxidant capacity Improved thermal stability Lowered tensile strength and elongation at break	[13]
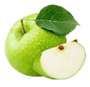 Green apple	Peel	Methylcellulose	Hot water extraction	Increased total phenolic content Improved antioxidant properties Lowered glass transition temperature Lowered tensile strength Increased elongation at break of the film	[122]
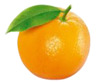 Mandarin orange 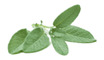 Sage leaf	Peel, leaf	Pectin	Homogenization	Enhanced antioxidant activity Increased total phenolic content Provided excellent physical properties Lowered thermal stability	[123]
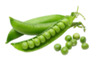 Pea 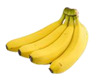 Banana	Peel	Guar gum	Ultrasound-assisted extracted	Exhibited good antioxidant capacity Improved moisture barrier Provided darker film with less transparency	[14]
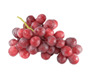 Red grape	Seed	*Ziziphora clinopodioides* essential oil, Chitosan, gelatin	Homogenization	Enhanced total phenolic content Provided excellent antimicrobial and antioxidant properties of the film Improved water vapor permeability Improved physical properties of the film	[124]
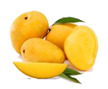 Mango	Peel	Fish gelatin	Homogenization	Demonstrated excellent antioxidant and antimicrobial activity Exhibited more rigid and less flexible film formation Decreased water vapor permeability	[125]
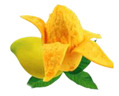 Mango	Kernels	Starch	Homogenization	Enhanced antioxidant and UV absorbing properties Decreased the visible light transparency of film	[126]
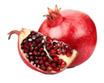 Pomegranate	Peel	Mung bean protein	Homogenization	Improved mechanical properties of mung bean protein film Enhanced antioxidant and antibacterial activity	[127]
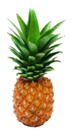 Pineapple	Peel	Pectin	Microwave heating extraction	Exhibited good antioxidant capacity Enhanced film opacity Decreased water vapor permeability Increased total phenolic content	[128]
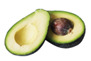 Avocado	Peel	Gelatin, carboxymethylcellulose	Homogenization	Improved antioxidant activity Inhibit the growth of *Rhizopus stolonifer* and *Aspergillus niger*	[129]
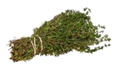 Thyme	Extract	Chitosan, starch	Homogenization	Provided great antioxidant activity Changed the microstructural and physical properties of film Enhanced mechanical response	[130]
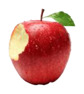 Apple	Extract	Cellulose, polyhydroxyalkanoate	Homogenization	Improved hydrophobicity and transparency Decreased the oxygen permeability of film Provided free radical scavenging capacity to film	[131]
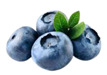 Blueberry, 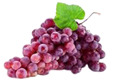 Red grape	Peel	Chitosan, carboxymethyl cellulose	Homogenization	Improved antioxidant activity Gives excellent oxygen barrier properties	[11]

**Table 4:** Usage of fruits and vegetables waste as antibrowning agents in food applications.

Sources of Waste	Part of the Plants	Targeted Plants	Extraction Method	Mechanisms of Action	Antibrowning Effects	References
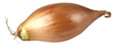 Shallots onion	Peel and stalk	Fresh-cut apples, Fresh-cut Potatoes	Conventional extraction	Inhibited activity of polyphenol oxidase	Controlled browning reaction Maintained the overall quality of apples and potatoes Extended shelf life	[141]
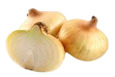 Borettana onion	Inner layers, juices and, distillates	Mushroom tyrosinase, potato slices, eggplant, fennel	Homogenization	Inactivated polyphenol oxidase via reduction of o-quinones to colorless o-diphenols	Reduced browning reaction Retained the color of plants	[142]
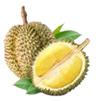 Durian	Shells	Fresh-cut asparagus lettuce	Liquid–liquid extraction	Inhibited both activities of monophenolase and diphenolase of tyrosinase	Delayed the development of surface browning Preserved the color of asparagus lettuce	[143]
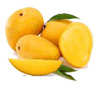 Mango	Peels	Potato puree	Homogenization	Inhibited activity of polyphenol oxidase	Inhibited browning reaction with inhibition values of 47.16%.	[144]
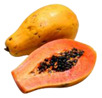 Papaya	Peels	Potato, apple, banana	Homogenization	Inhibited activity of polyphenol oxidase	Inhibited browning reactions in potato slices (59.87 + 0.77%), apple (21.08 + 1.94%) and banana slices (12.47 + 2.86%) through 6 h of storage	[145]
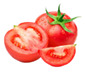 Tomato	Peel	Fresh-cut apples	Homogenization	Lowered activity of polyphenol oxidase	Controlled the browning reaction after 9 days at 5 °C	[146]
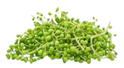 Unripe grapes	Juices	Dried “Golden Delicious” apple slices	Ethanolic extraction	Inhibited activity of polyphenol oxidase by unfolding the conformation of PPO enzyme structure and consequently decreasing catalytic activity	Reduced enzymatic browning Improved antioxidant capacity Preserved sensory quality of the fruits	[148]

**Table 5:** Removal or adsorbent capacities of targeted analytes using fruits and vegetables waste.

Waste	Targeted Analytes	Removal/Adsorption Capacity	Reference
Banana peel	Parathion	50.34%,	[148]
	Diazinon	63.86%	
Banana peel	Methylene blue	232.5 mg/g	[149]
Banana peel	Pb^2+^	57.1 mg/g	[150]
	Cd^2+^	46.9 mg/g	
Tangerine seed	Bendiocarb	7.97 mg/g	[151]
	Metolcarb	9.11 mg/g	
	Isoprocarb	13.95 mg/g	
	Pirimicarb	39.37 mg/g	
	Carbaryl	44.64 mg/g	
	Methiocarb	93.46 mg/g	
Orange peel	Chlorophenol	77.5%	[152]
Orange peel	Hg^2+^	0.25 ppm	[153]
Date stones	Profenofos	55.0%	[154]
Peach stone	Imidacloprid	PSAC 300: 80%, PSAC 500: 99%	[155]
Black and green olive stones	Methylene blue	Black ACOS: 714 mg/g, Green ACOS: 769 mg/g	[156]
Rambutan peel	Organochlorine	Fe^0^-B_Che_: 5–13%, Fe^0^-B_RtP_: 89–92%	[157]
*Citrus limetta* peel	Methylene blue	227.3 mg/g	[158]
Soy protein	Methylene blue	454 mg/g	[159]
Almond shells	Crystal Violet (CV) dye	1.075 mg/g	[160]

## Data Availability

Not applicable.

## Abbreviations

FAO—Food and Agriculture Organization; FVBP—fruit and vegetable by-products; TPC—total polyphenols content; DRBP—dried red beetroot peel; HVED—high-voltage electrical discharges; PEF—pulsed electric field; UAE—ultrasound-assisted extraction; GAE—gallic acid equivalent; CUPRAC—cupric reducing antioxidant capacity; DPPH—2,2-diphenyl-1-picryl-hydrazyl-hydrate; FRAP—ferric reducing antioxidant power; ABTS—2,2′-azino-bis(3-ethylbenzothiazoline-6-sulfonic acid); PLE—pressurized liquid extraction; SFE—supercritical fluid extraction; SPE—solid phase extraction; SC-CO_2_—supercritical CO_2_ extraction; NDES—natural deep eutectic solvents; ANN—artificial neural networking; RSM—response surface methodology; HPLC ESI-MS/MS—high-performance liquid chromatography/electrospray ionization tandem mass spectrometry; MAE—microwave-assisted extraction; LC-MS/MS—liquid chromatography with tandem mass spectrometry; HPLC—high-performance liquid chromatography; TCR—total carotenoid recovery; DSWE—date fruit syrup waste extract; ROS—reactive oxygen species; GCH/LSE—guar gum/carboxymethyl cellulose incorporated with lychee shell waste extract; EGCG—epigallocatechin gallate; BBA—blueberry ash; CMC—carboxymethyl cellulose; PEE—pulsed electric extraction; PGP—pomegranate peel particles; PPP—pomegranate peel powder; SA—sodium alginate; CMC—carboxymethyl; SOWE—shallot onion waste extract; ECTs—extractable condensed tannins; OCPs—organochlorine pesticides; Fe0—zerovalent iron; TVB-N—total volatile basic nitrogen; cyclo-DOPA—cyclic-dihydroxyphenylalanine.
